# Comparative Analysis of Efficacy, Toxicity, and Patient-Reported Outcomes in Rectal Cancer Patients Undergoing Preoperative 3D Conformal Radiotherapy or VMAT

**DOI:** 10.3389/fonc.2017.00225

**Published:** 2017-09-20

**Authors:** Antonia Regnier, Jana Ulbrich, Stefan Münch, Markus Oechsner, Dirk Wilhelm, Stephanie E. Combs, Daniel Habermehl

**Affiliations:** ^1^Department of Radiation Oncology, Klinikum rechts der Isar, Technical University Munich, Munich, Germany; ^2^Department of Surgery, Klinikum rechts der Isar, TU München, München, Germany; ^3^Institute of Innovative Radiotherapy (iRT), Helmholtz Zentrum München, Neuherberg, Germany

**Keywords:** rectal cancer, neoadjuvant chemoradiation, 3D conformal radiotherapy, VMAT, patient reported outcomes

## Abstract

**Background:**

Locally advanced rectal cancer (LARC) patients are usually treated within a multimodal therapy regime, in which the tumor resection plays the major role. This treatment ideally includes 5-fluorouracile (5FU)-based chemoradiation (CRT) leading to significantly improved local control rates. Local therapy as radiotherapy (RT) is required to be adapted referring to side effects and efficacy. Purpose of this study is the comparison of dosimetric parameters, acute and late toxicity, and quality of life in terms of patient-reported outcome (PRO) in patients treated with VMAT or 3D conformal radiotherapy (3DCRT) for LARC.

**Methods:**

Pelvic RT for LARC was performed with a prescription dose of 45 Gy in 1.8 Gy per fraction, 50.4 Gy in 1.8 Gy per fraction, or 50 Gy in 2 Gy per fraction. Chemotherapy included 5FU or 5FU/Oxaliplatin or Capecitabine-based RT. Acute and late toxicity were evaluated *via* National Institute Common Terminology Criteria for Adverse Events version (CTCAE) v4.03 and the Scoring System Late effects of Normal Tissue. Quality of life was established *via* EORTC QLQCR29.

**Results:**

After a median follow-up of 38 months (VMAT) and 78 months (3DCRT) there was no significant difference in progression-free survival (*p* = 0,85) but a significant difference in overall survival (*p* = 0.032). Regarding dose–volume parameters, patients treated with VMAT plans had a lower V20 of the bladder than 3DCRT-treated patients (*p* = 0.004). VMAT plans can also reduce Dmean of the right (*p* = 0.002) and left (*p* < 0.001) femoral head. Acute side effects between the VMAT and 3DCRT patients showed no significant difference. But concerning long-term effects, VMAT-treated patients had a significant lower appearance of high grade anal incontinence (*p* = 0.032). Quality of life (PRO) showed no significant different between the patients except of hair loss and worrying about weight.

**Conclusion:**

VMAT treatment of LARC in preoperative CRT revealed a reduction of dose to organs at risk (OARs) as bladder and femoral heads. However, no changes in acute and long-term toxicity profiles were detectable. For late toxicity and quality of life data longer follow-up times are required.

## Introduction

Locally advanced rectal cancer (LARC) patients are usually treated within a multimodal therapy regime, in which the tumor resection plays the major role. This treatment ideally includes preoperative short-term radiotherapy (“5 Gy × 5 Gy”) or long-term 5-fluorouracile (5FU)-based chemoradiation (CRT) with both modalities leading to significantly improved local control rates (1, 2). Nevertheless, distant metastasis and overall survival were not affected by adding neoadjuvant therapies. Concerning the increased acute and especially long-term toxicity rates in preoperatively treated patients together with the wanting effect on survival, preoperative radiation therapy (RT) has raised concerns in the surgical community (3–5). Nevertheless, quite recently, the German CAO/ARO/AIO-04 Trial showed for the first time that adding oxaliplatin to the 5FU-based CRT improves disease-free survival even though toxicity rates are slightly elevated (6).

Hence local therapy as RT is required to be adapted referring to side effects and efficacy. Quite recently the standard planning method for RT of LARC was 3D conformal RT (3DCRT). In recent years, technical improvements resulted in modern radiation delivering techniques, such as intensity-modulated radiotherapy (IMRT) (7). By using different beam angles and varying intensities, this technique allows a high conformal dose application to the target with the possibility of reducing the dose to the organs at risk (OARs) (8–11). A special variant of IMRT is the volumetric modulated arc therapy (VMAT) (12, 13). By rotation of the irradiation beam during therapy, this technique uses all possible directions to achieve high conformity and dose sparing of the OAR. The dosimetric superiority of VMAT over 3DCRT and even fixed-beam IMRT was already demonstrated for several pelvic tumor indications (14, 15).

For LARC volumetric modulated arc therapy (VMAT) is used with excellent results concerning target volume coverage and OAR sparing (16, 17). Also differences were found in acute and late toxicity compared to 3DCRT treatment (9).

Purpose of this study is the comparison of dosimetric parameters, acute and late toxicity and quality of life in terms of patient-reported outcome (PRO) directly reported by the patient *via* questionnaire in patients treated with VMAT or 3D conformal radiotherapy (3DCRT) for LARC.

## Patients and Methods

Between 2007 and 2014 a total of 85 LARC patients treated at our institution were suitable for this analysis. The diagnosis and clinical tumor stage was assured *via* histological sampling within a rigid rectoscopy and radiological imaging. 48 patients received VMAT which was introduced in our clinic 2010, and 37 patients received 3DCRT. The data of the patients and treatment plan characteristics, acute and late toxicity were collected retrospectively. Furthermore, EORTC QLQ–CR29 was established. All patients were treated with neoadjuvant CRT. CRT included 5FU or 5FU and oxaliplatin or Capecitabine-based (CAP). Patients obtained total mesorectal excision, partial mesorectal excision, abdominoperineal resection, intersphinctere rectal extirpation, or Hartmann surgery. Adjuvant chemotherapy was applied to 47 patients (28 patients of the VMAT group and 19 patients of the 3DCRT group).

The TNM classification of the sixth edition was used for the tumor staging of the patients (18). The tumor regression grading was rated by the quantification of the ratio of tumor tissue versus fibrotic tissue (Dworak score), or by the estimation of the percentage of vital tumor tissue in relation to the macroscopically identifiable tumor bed that was evaluated histologically (Becker score) (19, 20).

The Ethics Committee at the Technical University of Munich approved the study and patients gave informed consent in written form in terms of the QoL-questionnaire. There was no significant difference in the baseline characteristics including sex, T-category, positive lymph nodes, tumor localization, and cranio-caudal tumor extension (Table 1).

**Table 1:** Baseline characteristics.

Patient characteristics		VMAT		3DCRT		*p*-Value
		*n* = 48		*n* = 37		
			Median (IQR 25–75)		Median (IQR 25–75)	

Sex (% of male)		58		59		1.00

Age (years)			60 (53–69)		66 (64–71)	0.004

Primary tumor extension						
	cT1	0%		0%		0.415
	cT2	2%		8%		
	cT3	88%		87%		
	cT4	10%		5%		

Lymph node extension						
	N−	13%		24%		0.165
	N+	85%		73%		
	NX	2%		3%		

Tumor site						
	Upper third	6%		11%		0.222
	Middle third	52%		65%		
	Lower third	42%		24%		

Tumor length (cm)			5 (4–6)		4 (3–6)	

Grading						0.592
	G1	8%		11%		
	G2	71%		65%		
	G3	13%		5%		
	G4	0%		0%		
	GX	8%		19%		

Simultaneous chemotherapy		100%		97%		0.435

*IQR, interquartile range; 3DCRT, 3D conformal radiotherapy; cT1–cT4, clinical size of the tumor according to the “TNM Classification of Malignant Tumors” as developed by the Union of International Cancer Control; cN+, percentage of patients with positive lymph nodes*.

### Chemoradiotherapy

Radiotherapy was performed with a prescription dose of 45 Gy in 1.8 Gy per fraction, 50.4 Gy in 1.8 Gy per fractions, or 50 Gy in 2 Gy per fraction. The clinical target volume (CTV) and the OAR were outlined on a planning CT scan. Planning CT and treatment of the patients were performed by prone positioning with full bladder. The CTV included the primary tumor and the mesorectal, presacral, and internal iliac lymph nodes. CTV was enlarged in all directions by 10 mm to define the planning target volume (PTV). Eclipse system (Varian Medical Systems) and Oncentra MasterPlan (Nucletron) were used for treatment planning. Conventional 3DCRT was used from 2007 to 2014, whereas VMAT almost displaced 3DCRT since 2010.

Treatment planning was performed according to ICRU50/62 recommendations. The isodose curve representing 95% of the prescribed dose had to encompass the entire PTV and the maximum dose to the PTV was limited to <107% of the prescribed dose. To minimize the dose to the OAR, the following constraints were used: bladder: V40Gy < 50%; small bowel D50Gy < 10 cm^3^ and D40Gy < 100 cm^3^. VMAT was realized using 2–3 arcs and photon energies of 6 or 15 MeV. 3DCRT was planned with 4–8 coplanar beams. In all patients image-guided RT was conducted according to our internal guidelines.

The simultaneous chemotherapy for the patients included 5FU 250 mg/m^2^ or CAP 850 mg/m^2^ continuously or 5FU 1,000 mg/m^2^ on days 1–5 and 29–33 or 5FU 250 mg/m^2^ on days 1–14 and 29–35 and oxaliplatin 50 mg/m^2^ on day 1, 8, and 29.

### Tumor Response and Toxicity Evaluation

#### Toxicity

Acute and late toxicity were assessed *via* the National Institute Common Terminology Criteria for Adverse Events version (CTCAE) v4.03 and the Scoring System Late effects of Normal Tissue (21, 22). After a median follow-up of 47 months all surviving patients were contacted and asked for long-term effects.

#### Statistics

Statistical analyses included comparison of baseline parameters, side effects and different dose parameters using the Chi-Square test, Wilcoxon–Mann–Whitney *U* test and Fisher’s exact test. Overall survival and progression-free survival where compared using the log-rank-test. A *p*-value < 0.05 was considered as statistically significant.

## Results

### Dosimetric Comparison

Regarding dose distribution to the femoral heads, patients who were treated with VMAT had a lower mean dose of the right femoral head (*p* < 0.001) and of the left femoral head (*p* = 0.002) (Table 2). Concerning the bladder dose, patients achieving VMAT had a lower V20 (*p* = 0.004) compared to 3DCRT treated patients, whereas there was no difference between the group regarding the V30 (*p* = 0.419) and V40 (*p* = 0.334) of the bladder. The V10, V20, V30, and V40 of the bowel were not significant differing between the groups (V10: *p* = 0.306), (V20: *p* = 0.584), (V30: *p* = 0.496), and (V40: *p* = 0.960).

**Table 2:** Dose parameters.

	VMAT	3D conformal radiotherapy	
	
	*n* = 48	*n* = 37	*p*-Value
**Bladder**			
Dmean (Gy)	36.32	37.50	0.709
V20	91.85%	98.17%	0.004
V30	74.16%	69.69%	0.419
V40	49.66%	53.58%	0.334
**Bowel**			
V10	81.41%	67.16%	0.306
V20	63.50%	55.92%	0.584
V30	39.51%	32.86%	0.496
V40	20.29%	17.54%	0.960
**Left femoral head**			
Dmean (Gy)	25.21	29.40	0.000018
**Right femoral head**			
Dmean (Gy)	25.23	28.76	0.002

Chemoradiation was followed by surgery in all patients of the VMAT group and all patients of the 3DCRT group. Surgery was done after a median time of 6 weeks in both groups. A complete resection was achieved in 92% (VMAT) and 78% (3DCRT). 2% (VMAT) had R1 resection, 3% (3DCRT) R2 resection. RX resection occurred in 6% (VMAT) and 19% (3DCRT).

**Figure 1:**
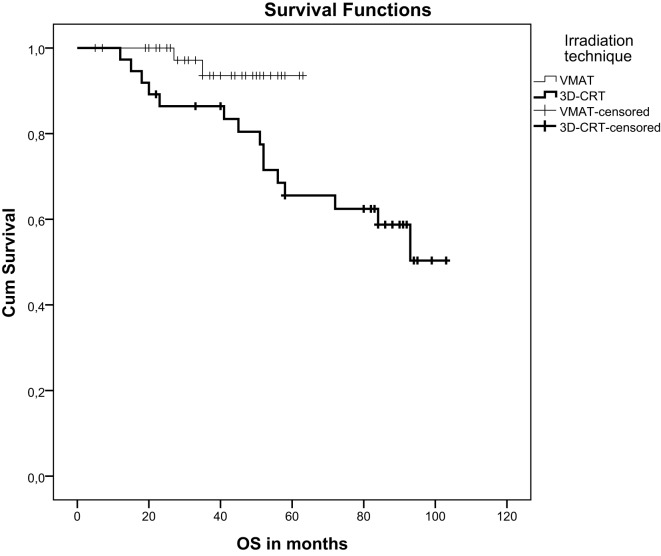
Kaplan-Meier curve demonstrating overall survival after VMAT or 3D-conformal chemoradiation.

**Figure 2:**
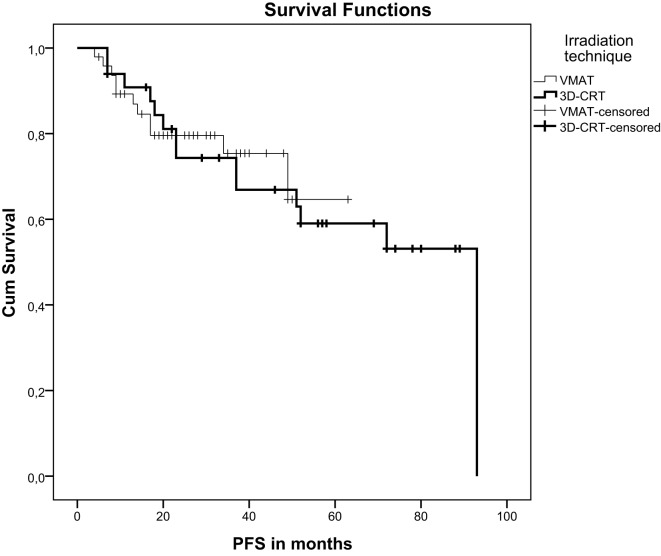
Kaplan-Meier curve demonstrating progression-free survival after VMAT or 3D-conformal chemoradiation.

Local failure was seen in one patient (2%) in the VMAT group and in two patients (5%) in the 3DCRT group. After a median follow-up of 38 months (VMAT) and 78 months (3DCRT) there was no significant difference in progression-free survival (*p* = 0.85) but a significant difference in overall survival (*p* = 0.032). Mean overall survival of VMAT-treated patients was 61 months and of 3DCRT patients 78 months (Figure 1). 3DCRT patients showed a median progression-free survival of 93 months. For VMAT patients the median endpoint of progression-free survival has not been reached yet (Figure 2).

In the VMAT patient group a total of 10 patients (21%) developed distant metastasis during the follow-up period. In 9 out of 10 patients, no local recurrence was detected. In the 3D-group, there were eight patients with detected distant metastases (24%). The reported two patients in the 3D-group with locally recurrent tumors also had distant metastasis during the observation period.

### Acute Side Effects

Acute side effects between the VMAT and 3DCRT patients showed no significant difference (Table 3).

**Table 3:** Acute side effects.

Side effects	VMAT	3D conformal radiotherapy	
	
	*n* = 48	*n* = 37	*p*-Value
Skin			0.281
I°	36%	33%	
II°	33%	46%	
III°	9%	15%	
IV°	0%	0%	
Diarrhea			0.523
I°	37%	22%	
II°	32%	34%	
III°	24%	34%	
IV°	0%	3%	
Change of the texture of the feces and existence of mucus or blood			0.133
I°	40%	23%	
II°	37%	50%	
III°	2%	13%	
IV°	0%	0%	
Anal incontinence			0.206
I°	13%	24%	
II°	13%	24%	
III°	0%	14%	
IV°	0%	0%	
Daily frequency of micturition			0.668
I°	38%	27%	
II°	9%	8%	
III°	0%	0%	
IV°	0%	0%	
Nausea			0.913
I°	38%	29%	
II°	17%	14%	
III°	8%	10%	
IV°	0%	0%	

Appeared adverse effects were diarrhea, change of the texture of the feces and existence of mucus or blood, anal incontinence, change of daily frequency of micturition, nausea, and skin side effects.

### Long-term Effects and Quality of Life

Patients were asked to answer EORTC QLQ–CR29 for late toxicity and quality of life to evaluate the PRO. As evaluating the questionnaire 96% of the patients treated with VMAT were alive and 59% of the 3DCRT group patients. The ratio of answered QLQ–CR29 was similar between both groups: 54% (VMAT) and 55% (3DCRT). The average time from RT treatment until responding the questionnaire was 37 months (VMAT group) and 81 months (3DCRT patients).

Between both groups there was no significant difference concerning long-term effects of the radiochemotherapy. But comparing high grade toxicity (grade-3 and -4) of long-term side effects, VMAT treated patients had a significant lower appearance of anal incontinence (*p* = 0.032) (Table 4).

**Table 4:** Long-term effects.

Side effects	VMAT	3D conformal radiotherapy	*p*-Value

	*n* = 48	*n* = 37	
Skin			0.296
I°	6%	14%	
II°	2%	3%	
III°	0%	3%	
IV°	0%	0%	
Diarrhea			0.245
I°	23%	5%	
II°	15%	16%	
III°	8%	14%	
IV°	4%	0%	
Obstipation			0.378
I°	4%	8%	
II°	0%	0%	
III°	2%	3%	
IV°	2%	3%	
Anal incontinence			0.067
I°	8%	0%	
II°	17%	11%	
III°	4%	16%	
IV°	0%	0%	
Anal incontinence high grade	4%	16%	0.032
Abdominal pain			0.794
I°	10%	8%	
II°	12%	8%	
III°	6%	0%	
IV°	0%	0%	
Urinary incontinence			0.077
I°	2%	5%	
II°	0%	11%	
III°	4%	3%	
IV°	0%	0%	
Urinary retention			0.741
I°	0%	3%	
II°	6%	3%	
III°	2%	0%	
IV°	0%	0%	

Concerning frequency of micturition, urinary incontinence, pain during micturition, abdominal pain, rectal/anal pain, distended belly, blood in stool, mucus in stool, dry mouth, problems with sense of taste, worried about health in the future, feeling less feminine/masculine or physically attractive as a result of disease or treatment, dissatisfaction with body, existence of or problems with a stoma bag, problems with bowel or stool passage, and difficulty getting an erection or having pain during intercourse no difference was observed (Table 4). Regarding hair loss and worrying about weight differences were determined (Table 5).

**Table 5:** EORTC QLQ–CR29.

EORTC QLQ–CR29	VMAT	3D conformal radiotherapy	*p*-Value

	*n* = 25	*n* = 12	
Hair loss			0.016
I°	79%	75%	
II°	21%	0%	
III°	0%	25%	
IV°	0%	0%	
Worried about weight			0.039
I°	60%	27%	
II°	36%	36%	
III°	4%	27%	
IV°	0%	9%	

## Discussion

Purpose of the study was the evaluation of dosimetric differences in a real-life patient cohort undergoing CRT with VMAT or 3DCRT and a possible correlation of dosimetric parameters with distinct toxicities and quality of life. For LARC the critical OARs are the bladder, the femoral heads and the bowel. Concerning the bladder, patients treated with VMAT plans had a lower V20 than 3DCRT treated patients (*p* = 0.004). VMAT plans can also reduce Dmean of the right (*p* = 0.002) and left (*p* < 0.001) femoral head. Concerning toxicity, our study showed a reduced long-term high grade toxicity of anal incontinence in VMAT-treated patients (*p* = 0.032).

A previous report from Xu et al. found that there is no correlation between dose–volume parameters of small bowel (V5–V40) and the acute lower gastrointestinal toxicity during preoperative concurrent CRT for rectal cancer patients when IMRT is used (23). The acute lower gastrointestinal toxicity is mainly influenced by rectal tumor volume. Our results showed similar dose parameters of the bowel (V10–V40) and also there was no difference between VMAT and 3DCRT patients concerning acute bowel toxicity.

Small bowel protection of patients with rectal cancer treated with IMRT can be achieved by prone positioning. Koeck et al. showed a significant dose reduction (up to 41%) for the small bowel of patients in prone position in the high and intermediate dose region, compared with the supine position (24).

High grade toxicity of anal incontinence in VMAT treated patients was reduced compared to 3DCRT patients. Arias et al. showed that the sphincter function (measured *via* Wexner score) in patients with LARC treated with preoperative radiochemotherapy was significantly less in those patients with V20 > 0 of the anal sphincters compared to those for which V20 = 0 (25). Hence dependent on tumor localization and distention, it could be important to reduce radiation dose of the anal sphincters and as well rectal surgery should be performed restricting sphincter function damage considering R0 resection.

Richetti et al. compared rectal cancer patients treated either with volumetric modulated arc therapy or 3DCRT with regard to dosimetric features (16). Patients treated with VMAT plans had a reduced Dmean and D1% of the femurs. There was no significant difference of Dmean or V40 of the bladder. This is consistently with our results. For normal tissues they also observed a lower integral and mean dose.

Dröge et al. demonstrated an improvement with volumetric modulated arc therapy comparing VMAT and 3DCRT high dose levels with regard to small bowel and the urinary bladder (9). For the small bowel the V40 was 28.4% with VMAT plans and 41.8% with 3DCRT plans. For the urinary bladder the V40 was 66.5% with VMAT-treated patients and 88.4% with 3DCRT-treated patients. As small bowel and colon complications arise after organ exposure of ≥50 Gy (small bowel) and ≥55 Gy (colon), this might explain that long-term toxicity for the bowel including proctitis was not significantly different between the VMAT and 3CRT group. This is supported by our study results which showed no difference comparing late toxicity like diarrhea and obstipation between both modalities. Dröge et al. who recently analyzed patients with advanced rectal cancer comparing volumetric modulated arc therapy and 3DCRT demonstrated a reduced high grade acute toxicity of skin reaction of volumetric modulated arc therapy treated patients (7% skin reaction ≥grade-3 in the 3DCRT group and 0% in the VMAT group) (9). Additional acute ≥grade-3 proctitis was significantly more frequent in the 3DCRT group (12% proctitis ≥grade-3 in the 3DCRT group and 2% in the VMAT group). Considering acute toxicities like enteritis, cystitis, and balanitis, VMAT- and 3DCRT-treated patients showed no differences. Analyzing late toxicity there were no differences regarding skin toxicity, proctitis, and cystitis between the VMAT and 3DCRT patients. In multi-group comparison, high grade late organ toxicity was significant more frequent in the 3DCRT group. As life expectancy after the treatment of patients suffering from rectal cancer is still long, a reduction of late toxicity is very important, particularly with regard to anal and urinary incontinence, impotence, and defecation irregularity.

The advantage of VMAT/IMRT over conventional 3DCRT concerning target dose conformity and reduction of higher doses to the OARs was already shown for different cancer subtypes as prostate cancer (15), gastric cancer (26), esophageal cancer (27), pancreatic cancer (28), and LARC (9, 16, 17). Furthermore, VMAT has the advantage of a reduction of treatment time delivery compared to 3DCRT application. The measured treatment time, defined as the time needed to deliver a single fraction with the exclusion of time needed to position the patients and to acquire data for image guidance is important. Richetti et al. showed a reduction of treatment time with VMAT at about 40% in contrast to 3DCRT (16). Therefore, VMAT potentially has the benefit of minimizing intrafractional errors. However, even though many advantages are obvious, there also was doubt with regard to the increased integral dose to the body by using IMRT-techniques. This might be of special interest in rectal cancer patients because long-term survival is expected in most patients undergoing neoadjuvant CRT, thus increasing the risk for a subsequent secondary cancer (29). Therefore, Zwahlen et al. investigated second cancer risk after RT for rectal cancer (11). They compared 3DCRT and VMAT. No significant difference for second cancer risk was detected between the different RT techniques.

Concerning oncological outcomes, there was clearly no significant difference regarding progression-free survival but there was a significant difference regarding overall survival. As VMAT was introduced into clinical practice recently, both groups represent differences in the follow-up periods. Furthermore, a limitation of the study is the small patient number. Concerning overall survival, late toxicity and quality of life data a follow-up of at least 10 years is desirable to evaluate if VMAT is truly superior in terms of reducing long-term sequelae.

## Conclusion

VMAT treatment of LARC in preoperative CRT revealed a reduction of dose to OARs as bladder and femoral heads. However, no changes in acute and long-term toxicity profiles were detectable. For late toxicity and quality of life data longer follow-up times are required.

## Ethics Statement

This study was carried out in accordance with the recommendations of the Ethikkommission der Fakultät für Medizin der Technischen Universität München with written informed consent from all subjects. All subjects gave written informed consent in accordance with the Declaration of Helsinki. The protocol was approved by the Ethikkommission der Fakultät für Medizin der Technischen Universität München.

## Author Contributions

AR, SM, MO, DW, SC, and DH were responsible for patient treatment and care. AR, JU, and DH collected the patients’ data. SM performed all statistical analyses. AR and JU drafted the manuscript. AR, JU, SM, MO, DW, and SC contributed to the analysis of data and revised the manuscript. DH conceived the study, helped to write, and finalized the manuscript. All authors helped with the interpretation of the data and read and approved the final manuscript.

## Conflict of Interest Statement

The authors declare that the research was conducted in the absence of any commercial or financial relationships that could be construed as a potential conflict of interest.

## Abbreviations

VMAT, volumetric modulated arc therapy; CRT, chemoradiotherapy; LARC, locally advanced rectal cancer; 3DCRT, 3D conformal radiotherapy; TME, total mesorectal excision; PME, partial mesorectal excision; APR, abdominoperineal resection; IPR, intersphinctere rectal extirpation; RT, radiotherapy; IMRT, intensity-modulated radiotherapy; CTV, clinical target volume; PTV, planning target volume; TRG, tumor regression grading.
